# Crossing of the Cystic Barriers of *Toxoplasma gondii* by the Fluorescent Coumarin Tetra-Cyclopeptide

**DOI:** 10.3390/molecules26247506

**Published:** 2021-12-11

**Authors:** Céline Dard, Baptiste Leforestier, Flaviane Francisco Hilário, Mohamed Dit Mady Traoré, Marie-Ange Lespinasse, Basile Pérès, Marie-Carmen Molina, Rossimiriam Pereira de Freitas, Anne Milet, Danièle Maubon, Yung-Sing Wong

**Affiliations:** 1 Team Host-Pathogen Interactions and Immunity to Infection, Institute for Advanced Biosciences, INSERM, CNRSINSERM U1209, CNRS UMR 5309, Univ. Grenoble Alpes, 38000 Grenoble, France; Celine.Dard@efs.sante.fr (C.D.); DMaubon@chu-grenoble.fr (D.M.); 2 Team SITH, CNRS UMR 5250, Univ. Grenoble Alpes, CNRS, DCM, 38000 Grenoble, France; baptiste.leforestier@univ-grenoble-alpes.fr (B.L.); anne.milet@univ-grenoble-alpes.fr (A.M.); 3 Team COMET, CNRS UMR 5063, Univ. Grenoble Alpes, CNRS, DPM, 38000 Grenoble, France; flavianehilario@ufop.edu.br (F.F.H.); mohamedditmadytraore@yahoo.fr (M.D.M.T.); marie-ange.lespinasse@univ-grenoble-alpes.fr (M.-A.L.); basile.peres@univ-grenoble-alpes.fr (B.P.); marie-carmen.molina@univ-grenoble-alpes.fr (M.-C.M.); 4 Departamento de Química, Universidade Federal de Minas Gerais, Av Pres Antônio Carlos, 6627, Pampulha, Belo Horizonte 31270-901, MG, Brazil; rossipfreitas@gmail.com

**Keywords:** cyclopeptides, histone deacetylase, *Toxoplasma gondii*, bradyzoite, cell-permeable agent, molecular dynamics

## Abstract

FR235222 is a natural tetra-cyclopeptide with a strong inhibition effect on histone deacetylases, effective on mammalian cells as well as on intracellular apicomplexan parasites, such as *Toxoplasma gondii,* in the tachyzoite and bradyzoite stages. This molecule is characterized by two parts: the zinc-binding group, responsible for the binding to the histone deacetylase, and the cyclic tetrapeptide moiety, which plays a crucial role in cell permeability. Recently, we have shown that the cyclic tetrapeptide coupled with a fluorescent diethyl-amino-coumarin was able to maintain properties of cellular penetration on human cells. Here, we show that this property can be extended to the crossing of the *Toxoplasma gondii* cystic cell wall and the cell membrane of the parasite in its bradyzoite form, while maintaining a high efficacy as a histone deacetylase inhibitor. The investigation by molecular modeling allows a better understanding of the penetration mechanism.

## 1. Introduction

The development of new therapies against infectious agents requires several prerequisites. While being effective against a given molecular target, usually through a high binding affinity, a drug candidate must also have adequate pharmacokinetic properties to reach that target, especially when compartmentalized within a cell. In addition to the lipophilic cell membrane, compounds are often required to cross other types of physical barriers with distinct properties. The intracellular parasite *Toxoplasma gondii* (*Tg*), under the immune system pressure, undergoes a conversion from the proliferative tachyzoite form to the cyst-enclosed quiescent bradyzoite [1]. This step converts the parasitic vacuole membrane surrounding the parasitic cells into a cystic cell wall. This is characterized by an enrichment in chitin, glycoprotein, and glycolipid to obtain a cyst cell wall with a thickness ranging from 250 to 750 nm [2]. This new barrier protects the bradyzoites from the external environment and helps escape the immune system defenses, making them persistent in the host. This conversion from tachyzoite and bradyzoite is reversible and can lead to a life-threatening disease in immunocompromised patients [3,4]. An active compound able to treat bradyzoite must therefore both cross this richly glycosylated cystic wall and pass through the lipophilic cell membrane of the parasite. This makes this model particularly useful for studies of compound penetration through different cell barriers.

In the case of the crossing of lipid membranes by compounds, Lipinski’s rules are commonly applied to predict the permeability of molecules, which depends on a few criteria (the Lipinski’s rule of five, Ro5 [5]), such as a molecular weight limited to less than 500 Da. However, little is known about rules that dictate the crossing of a cystic cell wall. Using charged fluorescent molecules, Lemgruber showed that the ability of molecules to pass through and diffuse into the cyst matrix depends on their size and charge (compounds negatively charged up to 960 Da) [2].

**Figure 1:**
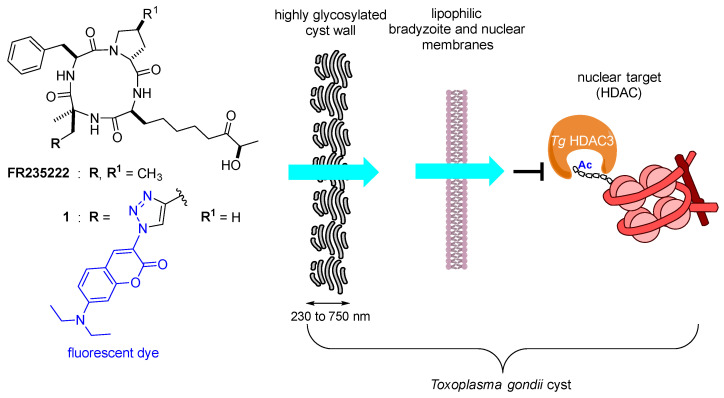
Natural product FR235222 and its fluorescent coumarin triazole analogue **1**. In *T. gondii*, the cyst wall, the bradyzoite external membrane, and its nuclear membranes are the barriers that must be overcome for these compounds to reach their nuclear target, *Tg* HDAC3.

In the course of our work on deciphering epigenetic mechanisms, it was identified that the natural cyclic tetrapeptide FR235222 [6] was able to inhibit *Tg* histone deacetylase 3 (*Tg* HDAC3), located in the nucleus of the parasite, by strongly affecting the tachyzoite growth and by promoting its differentiation into bradyzoite [7]. Indeed, *Tg* has singularly evolved into a finely tuned and epigenetically regulated developmental program to operate stage conversion in response to environmental cues [8,9]. Moreover, the compound is also active on ex vivo cyst-enclosed bradyzoites [10], showing that it can cross the cystic cell wall and the bradyzoite membrane to reach its nucleus (Figure 1). Based on our previous work, this natural product owes it remarkable selectivity and cellular penetration to two main characteristics. The flexible aliphatic chain and the (*R*)-hydroxyl ketone zinc-binding group are in one part responsible for the selective inhibition of class I HDACs isoforms [11]. On the other hand, the constrained and uncharged cyclic tetrapeptide moiety confers cell penetration properties, as we have demonstrated on human cells [12]. This moiety is found in natural products [13], and the cell penetration properties can be attributed to its cyclic nature [14,15,16]. Indeed, such a structure could dynamically adopt hydrophobic or hydrophilic conformations depending on the polarity of the medium [17] and thus confer permeability properties that can go beyond the Ro5, especially in terms of molecular weight limitation [18,19,20,21]. In a previous study, we demonstrated that the cyclic tetrapeptide moiety found in FR235222 can transport Wang’s fluorescent coumarin triazole (in blue, Figure 1) [22], which has a low cell permeability, into the nucleus through the lipid bilayers [12]. The aim of this work is to demonstrate the ability of this cyclic tetrapeptide to transport this agent through two types of membrane, hydrophilic and hydrophobic membranes, using the *Tg* cyst as a model and to provide insights into the mechanism involved in its penetration by molecular modeling.

## 2. Results and Discussion

### 2.1. Evaluation of the Cyclic Tetrapeptide Penetration through the Cyst Wall and Bradyzoite Membranes

**Figure 2:**
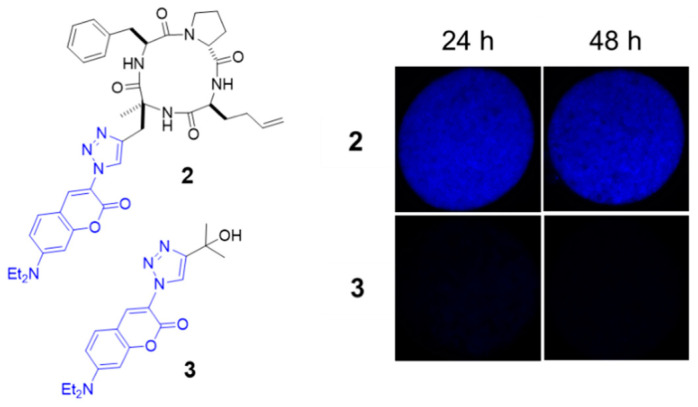
Comparison of cyst penetration observed by microscopy between the fluorochrome coupled with the cyclic tetrapeptide (compound **2**) and the dye alone (compound **3**); Ex vivo *T. gondii* cysts (PRU strain) incubated with the compounds **2** or **3** at concentration = 1 µM, 37 °C, exposure time = 100 ms.

To focus on the ability of the cyclic tetrapeptide moiety to transport compounds through the cyst wall, we began our investigation by monitoring the transport of the poorly cell-permeable fluorochrome **3** (Figure 2) by optical microscopy. The compound **2** comprising the fluorochrome attached to the cyclic tetrapeptide has already shown its ability to cross lipid membranes in human cells [12]. After incubation of isolated cysts with 1 µM of compounds for 24 h at 37 °C and followed by PBS washing, cysts did not show significant labeling with fluorochrome **3** alone whereas the compound **2** labeled them with a high intensity (exposure time for image capture = 100 ms).

**Figure 3:**
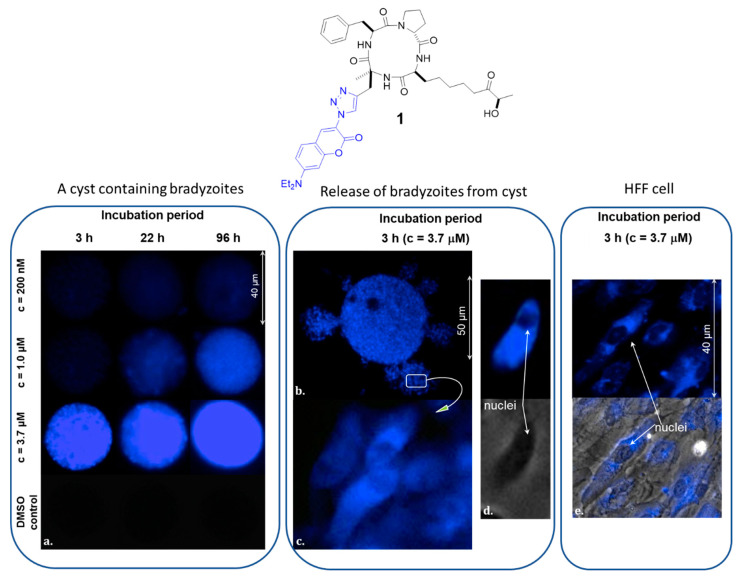
(**a**) Ex vivo *T. gondii* cysts incubated with compound **1** at a growing concentration (c = 200 nM to 3.7 µM) for 3, 22 and 96 h, 37 °C, exposure time = 100 ms; (**b**) Cyst treated with **1** (c = 3.7 µM for 3 h and released from the cyst, 37 °C, exposure time = 100 ms; (**c**,**d**) Bradyzoites at a higher magnification (×100); (**e**) HFF cells treated with **1** (exposure time = 100 ms).

To investigate whether the cyclic tetrapeptide was also able to cross the lipid membrane of the bradyzoite and reach its nucleus, we next used the fluorescent compound **1** [12] with the selective HDACi part that targets the parasite nucleus (Figure 3). After incubation of isolated cysts with different concentrations of **1**, increasing fluorescence was observed (from 3 h to four days), suggesting both penetration and accumulation of **1** in the cyst (Figure 3a). To ensure that the fluorescent compound was incorporated to the parasite cell, bradyzoites were released from treated cysts (Figure 3b). Microscopy images clearly show labeling of these bradyzoites by the fluorescent compound **1**, suggesting that **1** is not only confined to the cyst cell wall or the matrix of the cyst, but is also able to penetrate the cysts and reach the intracellular structure of the bradyzoite. At high magnification (×100), bradyzoites show diffuse fluorescence in their cytoplasm with higher brightness around the nucleus, as previously observed in human cells (Figure 3c,d) [12]. This labeling pattern was also confirmed on the host human cell, human foreskin fibroblast (HFF), used in our in vitro model (Figure 3e).

Interestingly, the fluorescent compound **1** retained its HDACi property on *Tg* tachyzoites and on human cells HFF (Table 1). 

**Figure 4:**
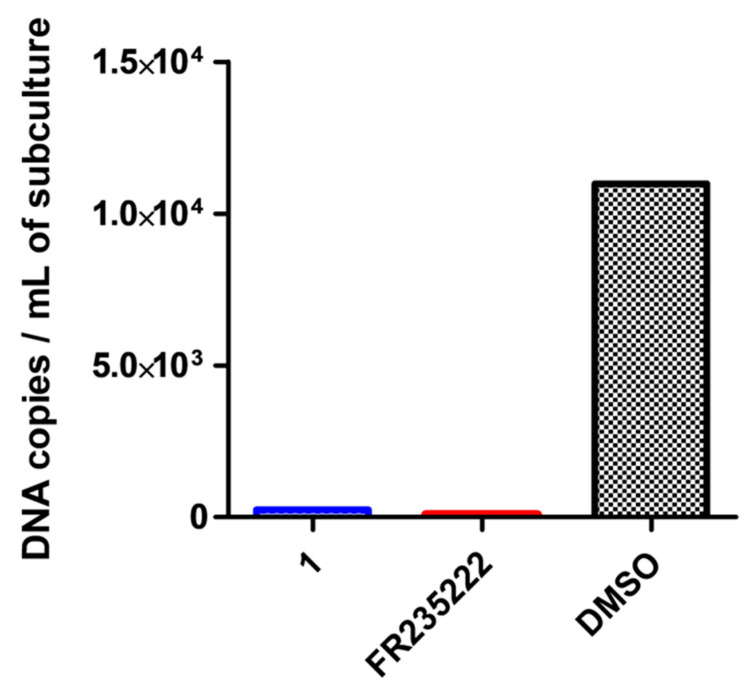
Fluorescent compound **1** is effective on ex vivo *T. gondii* cysts in in vitro assays.

To confirm that the compound does indeed reach its nuclear target (*Tg* HDAC3) in the cysts, its efficacy as an HDACi was also tested on the bradyzoite. FR235222 has previously been shown to be active on cysts ex vivo [10]. Following the same ex vivo protocol, we treated cysts for 7 days with **1** and FR235222. The total number of cysts did not differ from day 1 to day 7, suggesting that cysts were not destroyed during the incubation time with both compounds. At day 7, bradyzoite cells were released from the cysts and placed in a HFF monolayer subculture flask to trigger the tachyzoite interconversion. On day 14, this subculture was entirely harvested and subjected to a specific quantitative PCR targeting a *Tg*-specific gene [23]. We showed that treatment with **1** or FR235222 significantly reduced the parasite load of the subculture compared with the DMSO control (Figure 4). We concluded that the fluorescent compound **1** has the same inhibitory effect on cysts as FR235222. Of note, as for the tachyzoites, the presence of the fluorochrome did not significantly affect the anti-cystic activity of the active part of the compound **1**.

Prior treatment of ex vivo PRU strain cysts with **1** (c = 200 nM) or FR235222 (c = 200 nM) dramatically reduced the number of parasites (number of *Tg* DNA copies in the PCR sample) in parasite subculture compared to the control (DMSO). 

To demonstrate that the compound **1** specifically targets crucial nuclear HDAC3 of the parasite, we investigated its effect on a *Tg hadc3*-mutated strain. The HDAC-specific residue T99 of *Tg* HDAC3 plays a crucial role in the inhibition activity of FR235222 as point mutation leading to amino-acid substitutions T99A or T99I in *Tg* HDAC3 confers resistance to this product [7]. When tested on the RH *Tg hdac3* T99A strain, the compound **1** showed no effect on T99A tachyzoites’ proliferation while the growth of the RH wild-type strain was completely inhibited (Table 2).

**Figure 5:**
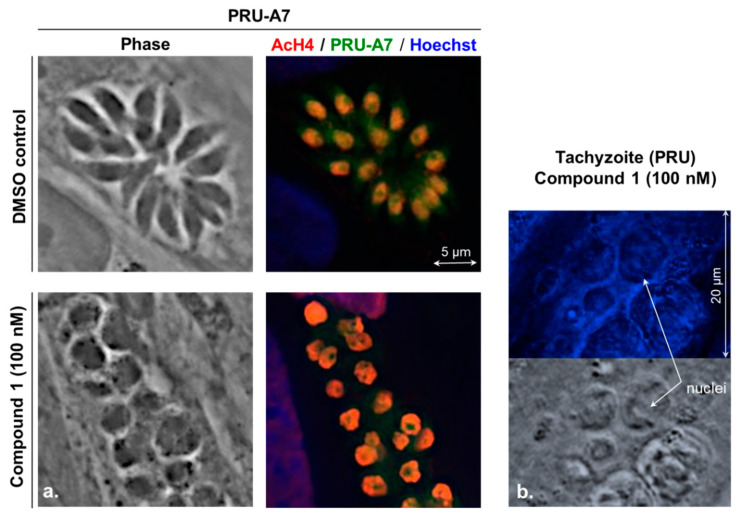
(**a**) In vitro tachyzoites PRU-Δhxgprt-A7 strain (PRU-A7) treated with the compound **1** results in an increased level of histone-H4 acetylation (AcH4) associated with dysmorphic features (increased size, giant polynucleated parasites, rounded shape); Fixed cells, AcH4 in red (anti-acetyl-H4 polyclonal rabbit antibodies (primary antibodies) (Millipore), followed by the secondary antibodies goat anti-rabbit IgG coupled with either Alexa Fluor 568 (Invitrogen) at a 1:1000 dilution), tachyzoite GFP protein (PRU-A7 in green)), HFF, and tachyzoite DNA in blue (Hoechst) (**b**) Localization of the compound **1** in treated PRU tachyzoites observed by microscopy (c = 100 nM, 48 h, exposure time 1000 ms).

Finally, the compound **1** and FR235222 also displayed the same microscopic phenotype on multiplying tachyzoites from different *Tg* strains (Prugnaud-A7-GFP) by inducing round-shaped polynucleated parasites (instead of fusiform mononucleated ones) and increased the histone-H4 acetylation level (Figure 5) [7].

### 2.2. Conformational Studies by NMR 

To achieve permeability to hydrophilic and hydrophobic barriers, we hypothesized that the cyclic tetrapeptide should easily adapt its polarity to the environment. There are several hypotheses regarding the passive diffusion modes of cyclopeptides across the lipid bilayer of cells. The first correlates membrane permeability with its ability to readily change cyclopeptide conformation by fluctuating the *cis*/*trans* isomerization between its amide functions to match the polarity of the medium. Thus, in an aqueous or polar medium, the most favorable conformation would be the “open” one, with flexible conformations that allow maximum amide bonds to interact with the medium. In a hydrophobic medium, the conformation of the cyclopeptide would change to the “closed” one to privilege the intramolecular hydrogen binding (IMHB) between the amide bonds [14,24]. This results in a conformation with a compact IMHB network in hydrophobic media compared to more diverse and flexible conformations in water and polar media. When the permeability is not related to a conformational change in the cyclopeptide framework [21,25], another hypothesis is based on the proper connection between the hydrophobic surfaces of the cyclopeptide, which can be an important criterion for increasing cellular permeability [25].

**Figure 6:**
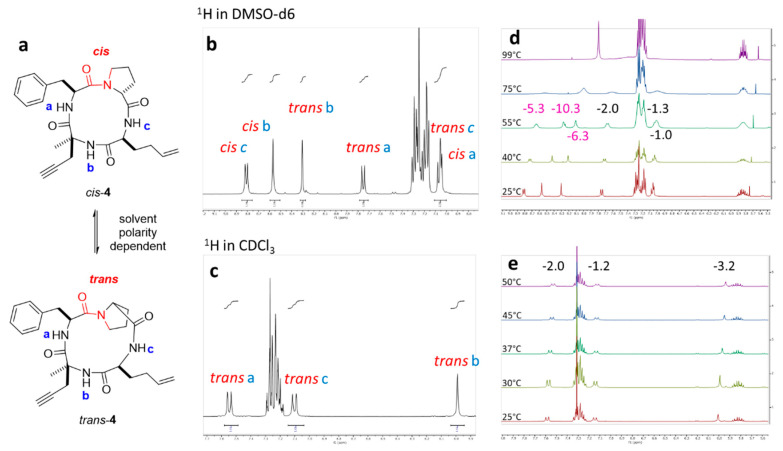
(**a**) Cyclic tetrapeptide **4** underwent proline *cis*/*trans* isomerization depending on the polarity of the media. In red, representation of the amide bond between the proline and the phenylalanine in *trans* and *cis* conformations; (**b**) and (**c**) ^1^H NMR amide protons of **4** in DMSO and CDCl_3_, respectively; (**d**) and (**e**) Temperature shift experiments of **4** in DMSO and CDCl_3_, respectively, with the Tc value in cyan higher than 4 ppb/K.

NMR studies by Bifulco and co-workers showed that the cyclic tetrapeptide with a D-configuration proline adopts *trans* and *cis* amide conformations between proline and phenylalanine in DMSO-d6 (Figure 6a), reflecting its conformation flexibility in polar solvent [17]. This flexibility suggests that the mode of entry could be related to solvent-dependent adaptation of the peptide ring configuration. Conformational computational studies performed in water by Oakley and co-workers have shown that cyclic tetrapeptide structures exhibit a facilitated conformational change in their *cis*/*trans* isomerization between the amide functions of two successive amino acids, due to the substantial distortion of the amide bonds imposed by the tension of the 12-membered rings [26]. Their calculations also showed that changing the polarity of the solvent influenced the conformation and the number of IMHBs. 

### 2.3. Conformational Studies by Molecular Modeling

To distinguish experimentally whether our cyclic tetrapeptide readily changes conformation according to the nature of the medium, NMR experiments in two different types of solvent, CDCl_3_ and DMSO-d6, were carried out on the simpler cyclopeptide **4** [12] to minimize interferences with other functional groups. We confirmed by ^1^H NMR (Figure 6b,c) and ROESY (Figure S4) experiments that **4** indeed displayed this *cis*/*trans* equilibrium in DMSO-d6, while only one conformer in *trans* conformation was visible in CDCl_3_ (NOESY, Figure S7). Temperature shift experiments allow the involvement of an amide proton (^1^H^N^) either in an intramolecular hydrogen-bond or in a more solvent-exposed interaction to be distinguished [27,28]. For the compound **4**, we observed significant ^1^H^N^ shifts in DMSO-d6 (<4 ppb/K are considered solvent exposed) for both *cis* and *trans* conformations, while those observed for the conformation in CDCl_3_ remained modest. In DMSO, the cyclic tetrapeptide **4** amide protons seem to be more exposed to solvent, especially at the Hb region (<6 ppb/K).

**Figure 7:**
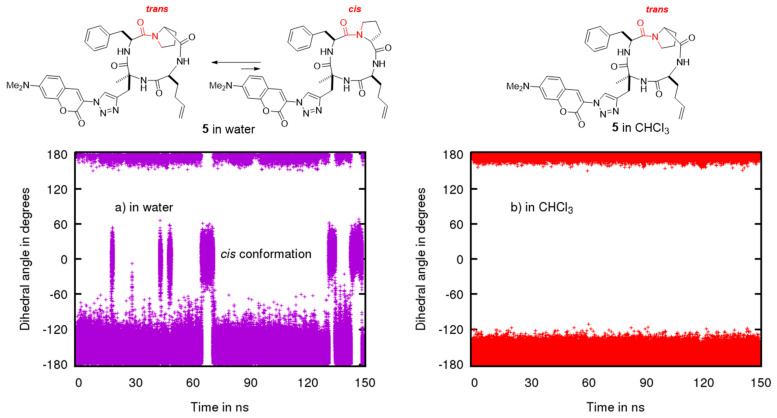
Evolution of the dihedral angle corresponding to the *cis/trans* isomerization by molecular dynamics (values close to zero correspond to the *cis* conformation and values close to 180° or −180° to the *trans* conformation) (**a**) in water and (**b**) in CHCl_3_.

To confirm the conformational flexibility of our cyclic tetrapeptide in a polar solvent and its preference to form a more compact conformation in a less polar medium, we performed calculations on the structure **5** (Figure 7) with a simplified coumarin part (the two ethyl groups are replaced by two methyl in order to keep a reasonable number of conformations and to focus on the cyclopeptide moiety). We used molecular dynamics (MD) simulation with Amber 18 [29] to generate stable conformers of **5** in CHCl_3_ and in water.

**Figure 8:**
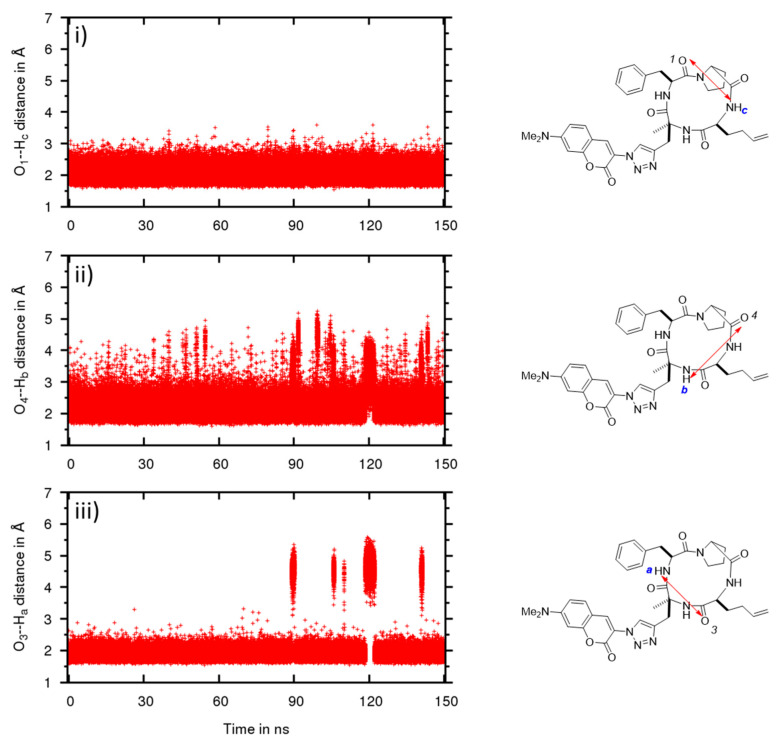
Evolution of the hydrogen bond distance between C=O and NH in compound **5** in CHCl_3_ by molecular dynamics simulation. Distances between (**i**) O1 and NH**c**, (**ii**) O4 and NH**b** and (**iii**) O3 and NH**a**.

**Figure 9:**
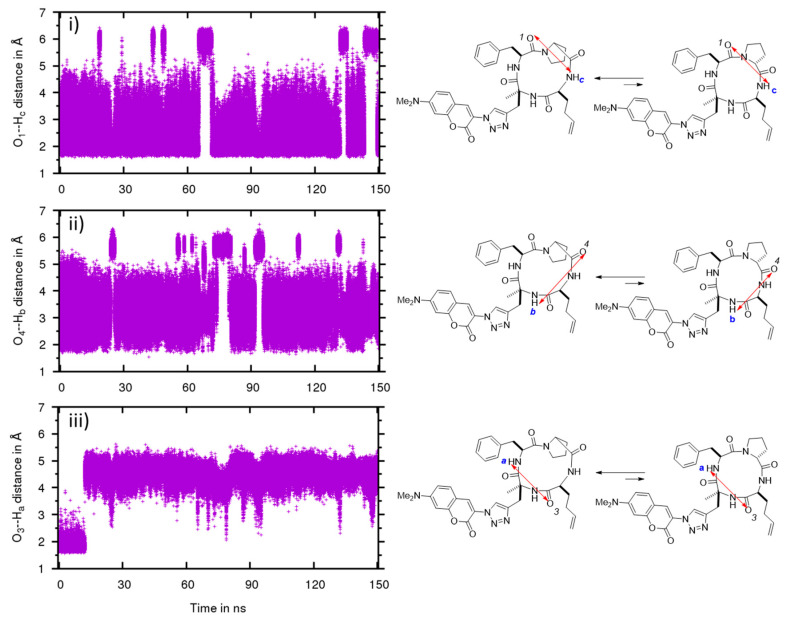
Evolution of the hydrogen bond distance between C=O and NH in compound **5** in water by molecular dynamics simulation. Distances between (**i**) O1 and NH**c**, (**ii**) O4 and NH**b** and (**iii**) O3 and NH**a**.

To analyze their conformational difference, the conformers obtained were described in terms of the dihedral angle of the amide bond between proline and phenylalanine (Figure 7) and representative IMHBs (Figure 8 and Figure 9).

In CHCl_3_, the MD confirmed that the cyclic tetrapeptide remained stable in the *trans* conformation (Figure 7b), which is also evidenced by the preservation of the IMHB between O1 and Hc (Figure 8i). The other two IMHBs sometimes reached the breaking point (Figure 8ii,iii) but remained mostly intact. These results confirm the stability of the cyclic tetrapeptide in low dielectric media, such as CHCl_3_, to adopt a *trans* amide conformation between proline and phenylalanine, with “closed” conformations that maximize their IMHBs. 

In water, MD simulations of the structure **5** showed more conformational flexibility with a variety of more open conformational states (Figure 9). Unlike in CDCl_3_, *cis* conformers can be formed in water and a more broader fluctuation band for *trans* conformers was observed (Figure 7a). Length variation and breakage of IMHBs were also more pronounced (Figure 9i–iii). A striking feature was the clear rupture of the IMHB between O3 and Ha (Figure 9iii), showing that these open water-interacting conformers are clearly more stable in this medium. To go into more detail on the conformational behavior and structural aspects of **5**, in CHCl_3_ (Figure 8), three IMHBs are highly present during the simulation. The hydrogen bond between O3 and N-Ha is present 91% of the time of the simulation with an average distance between the heavy atoms involved of 2.773 Å and an angle of 148.6°, the hydrogen bond between O1 and N-H**c** is present 47% (average distance of 2.781 Å and average angle of 142.9°) and the hydrogen bond between O4 and N-H**b** 43% of the time of the simulation (average distance of 2.841 Å and 143.1°). Other IMHBs are present but less than 0.3% of the time of the simulation. All these hydrogen bonds maintain the molecule in a « ball » form that maximizes the IMHB and prevent the *cis*/*trans* isomerization. The hydrogen bond between O1 and N-Hc never leads to the *cis* conformation and the value of hydrogen bond is always lower than 3 Å and always lower than 4 Å for the distance between the hetero atoms. The longer distances correspond to an accentuated stretch and not to a different conformation. On the contrary, most of the time, the hydrogen bond between O3 and N-Ha exhibits a short IMHB but when it achieves a higher distance, it reaches a new conformation with the hydrogen bond clearly broken and attains hydrogen bond distances of more than 4 Å. On the other hand, in water (Figure 9), the IMHBs are rare and only the hydrogen bond between O1 and N-H**c** is present 26% of the time of the simulation (average distance of 2.802 Å and average angle of 148.3°). It is quite interesting to note first the difference with the data obtained in CHCl_3_ and second the clear correspondence between the high value of this hydrogen bond (bond distance higher than 5 Å) and the *cis* conformation. The other IMHBs are rarely present (1% for O4 and 7% for O3) due to the competition with the formation of the hydrogen bond with water.

To obtain a deeper insight into the involvement of IMHBs, we collected the percentage of the presence of hydrogen bonds between the water and molecule **5** during the simulation (Table 3). The C=O bonds of the molecule are highly involved in hydrogen bonds with water, and on average, there are 7.6 bonds between the molecule and the solvent and only 0.40 IMHB. On average in CHCl_3_, we obtained 1.8 IMHB. All the C=O of the molecule are linked to one water molecule at least 50% of the time of the simulation and for 18% of the time of the simulation, they exhibit two hydrogen bonds with water. We can also note from Table 3 that O2, which is not involved in any IMHB in CHCl_3_, is the most involved group for the hydrogen bonds with water. 

**Figure 10:**
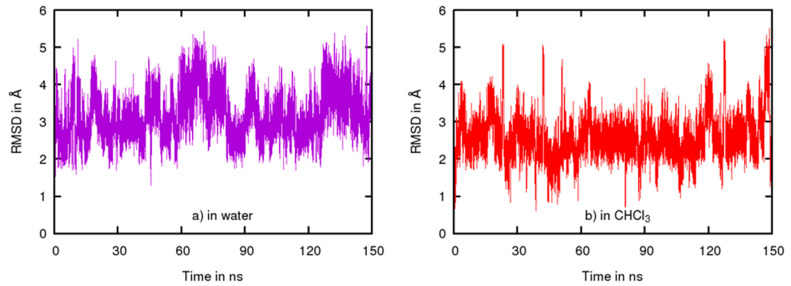
Evolution of the RMSD of compound **5** in Å (**a**) in water and (**b**) in CHCl_3_.

To estimate the magnitude of conformational changes in the compound **5**, we calculated the RMSD of the molecule along the trajectory using the first geometry of the trajectory as a reference (Figure 10). In water, the average value of the RMSD was found to be 3.147 Å with a standard deviation of 0.624 Å whereas in CHCl_3_, the average value of the RMSD was only 2.526 Å with a standard deviation of 0.572 Å, demonstrating a more flexible behavior of compound **5** in water compared to CHCl_3_.

Taken together, the MD simulations reveal the flexible nature of the cyclic tetrapeptide structure, which can readily adopt different conformations, closed in low dielectric media and more open in polar solvents (for representative structures, see Figures S9–S11).

### 2.4. Permeability between Low and High Dielectric Environment

**Figure 11:**
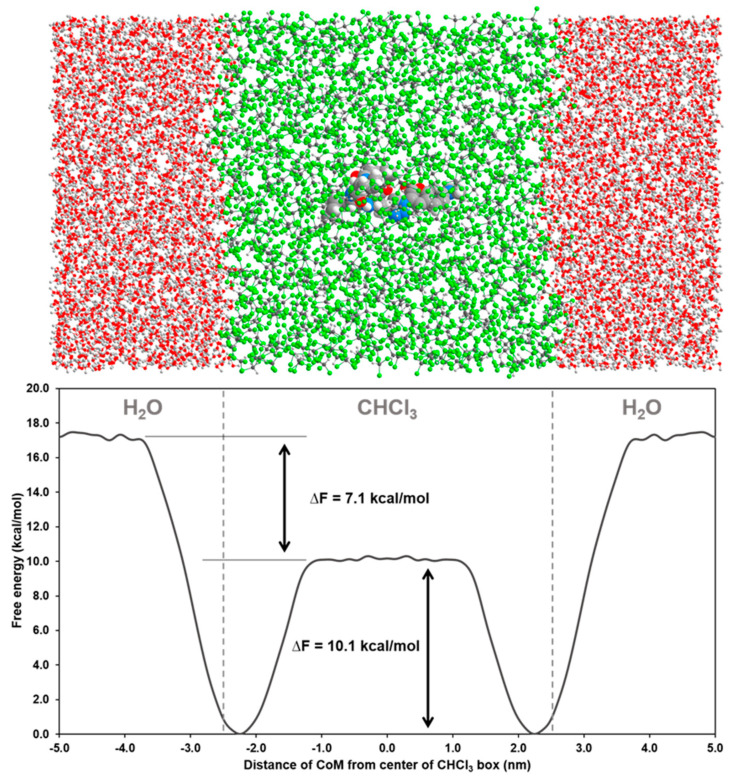
Free energy profiles of the compound **5** for its transit between water (red) and CHCl_3_ (green).

To streamline the permeability of our cyclic tetrapeptide, we computed the conformation-dependent partition free energy between low and high dielectric media [30,31,32,33]. A lower energy barrier to pass the two media is expected for a high permeability compound. A CHCl_3_ (low dielectric environment) and water (high dielectric environment) system has been used as a model to evaluate the passage of bioactive compounds and cyclopeptides across the lipid membrane [30,34]. In our case, it can also account for the crossing of the cyst wall barrier, which has both hydrophilic and hydrophobic properties. From the interface between water and CHCl_3_, we performed metadynamics to induce the transit of **5** and obtain its free energy profile (Figure 11). If we compare our results with those of Meng and Xu [31], we find very similar energy profiles for compounds that are cell permeable. No barrier for the transit of **5** between water and CHCl_3_ was found and, indeed, the energy is never higher than the energy in bulk water. The free energy profile first decreases from bulk water to CHCl_3_ until it reaches a minimum, then it increases to achieve an energy barrier of 10 kcal/mol in the center of the CHCl_3_ part. These results suggest that the molecule is able to easily adopt conformations that fit the environment in both solvents and also at their interface. These features may explain the ease of penetration of these compounds between a low and high dielectric environment. 

## 3. Conclusions

In this study, we showed that an analogue of the HDAC inhibitor FR235222 bearing a low cell permeability fluorescent coumarin triazole moiety retained its nanomolar efficacy in inhibiting *T. gondii* in its cystic form. This ability to transport the fluorescent probe and reach its nuclear target by crossing two types of membrane barriers, hydrophilic and hydrophobic, is related to its cyclic tetrapeptide moiety. Through NMR studies and molecular dynamics conformation simulations, we demonstrated that the cystic wall permeability of this scaffold is due to its chameleon-like behavior with its ease of conformational change allowing it to adapt to its environment. This easy conformational adaptation plays a role when crossing the two types of medium. The conformation-dependent partition free energy level of this cyclopeptide at the interface between the low and high dielectric media is consistent with a passive crossing from one compartment to the other. We are currently testing new cyclopeptide scaffolds to understand their ability to cross physiological barriers and transport compounds into the cell.

## 4. Materials and Methods

### 4.1. Biological Assay

#### 4.1.1. Parasites Strains 

The parasite strains used in this study are the following: the *T. gondii* type I RH strain that has lost the ability to complete cyst conversion, the *T. gondii* type II Prugniaud (Pru) strain that is capable of robust bradyzoite differentiation, the *T. gondii* type II Pru-Δhxgprt-A7 strain that expresses green fluorescent protein (GFP) [35], the Pru-Δku80 strain [36,37], and the RH *Tg hdac3* T99A strain (previously obtained in our laboratory). 

#### 4.1.2. In Vitro Parasites and Cell Culture

All *T. gondii* strains were maintained by serial passage in an HFF (Human Foreskin Fibroblasts) monolayer under tachyzoite growth conditions in Dulbecco’s modified Eagle medium (DMEM; Invitrogen) supplemented with 10% (vol/vol) fetal bovine serum (FBS; Invitrogen), 4 mM glutamine, 500 U/mL penicillin, 250 µg/mL streptomycin at 37 °C and 25 mM Hepes buffer (Invitrogen) in 5% CO_2_. 

#### 4.1.3. Mice

A total of 30 6-week-old female Swiss mice weighing about 18–22 g each from Janvier Laboratories (Le Genest-Saint-Isle, France) were used for all the ex vivo and in vivo experiments. Mouse care and experimental procedures were performed under pathogen-free conditions in accordance with established institutional guidance and approved protocols from the Institutional Animal Care and Use Committee of the University Grenoble Alpes (agreement n°B3851610006).

#### 4.1.4. Mice Infection

The mice were infected by oral gavage of infectious *T. gondii* cysts (Pru strain). To obtain the infectious cysts, the brains from chronically infected mice (>12 weeks with PRU strain) were crushed in PBS, the number of cysts was microscopically quantified, and the mice were force fed with 100 μL of brain homogenate containing 20 to 40 cysts using a ball-tipped feeding needle. Animal euthanasia was completed in an approved CO_2_ chamber.

#### 4.1.5. Cysts Purification

Cysts were isolated from the brains of mice chronically infected with the Pru-Δhxgprt-A7 or the Pru strains for at least 6 weeks, either using the Percoll gradient method as described previously [38] or directly harvesting the cysts using a 10-μL pipet for the dye experimentation in order to prevent deterioration of the cyst wall for the permeability studies. 

#### 4.1.6. Immunofluorescence Microscopy of Tachyzoites and Cysts

Grown *T. gondii* tachyzoites-infected HFF monolayers were fixed in 3% formaldehyde for 20 min at room temperature, permeabilized with 0.1% (v/v) Triton X-100 for 15 min, and blocked in PBS containing 3% (w/v) bovine serum albumin (BSA). The infected cells were then incubated for 1 h with the primary antibodies indicated in the figures followed by the addition of secondary antibodies conjugated to Alexa Fluor 488 or 594 (Molecular Probes) at a 1:1000 dilution for 1 h. The nuclei of both host cells and parasites were stained for 10 min at room temperature with Hoechst 33258 at 2 μg/mL in PBS. After four washes in PBS, coverslips were mounted on a glass slide with Mowiol mounting medium.

After treatment with the different compounds, cysts and bradyzoites were microscopically observed between the slide and the slip cover.

Images were acquired with a Axioplan 2 fluorescence microscope (Carl Zeiss Microimaging, Thornwood, NY, USA) and processed with the ZEN software (Zeiss).

#### 4.1.7. Drugs

The cyclic tetrapeptide FR235222 was provided by Astellas Pharma Inc. (Osaka, Japan). Compounds **1**–**4** were synthesized in our laboratory and their synthetic protocols were reported from previous works [12].

### 4.2. Computation

#### 4.2.1. Molecular Dynamics

They were performed using the Amber 18 software [29] to investigate the solvation pattern of the molecule **5** in water and in CHCl_3_ (these were used as mimics of the membrane to be crossed and the hydrophobic interactions found in this medium). The recently developed CREST method [39] was used to obtain reliable starting geometries for the dynamics in water using implicit models of solvation. Using these geometries, single point energy calculation at the HF/6-31G* was performed to obtain partial charge on each atom with the restrained electrostatic potential (RESP) method [40]. The force fields parameters for the molecule were then deduced using the GAFF [41] (generalized amber force fields) parameters with Amber 18. The molecule was then immersed in a cubix box of TIP3P waters or CHCl_3_, adding 4867 and 1047 molecules of solvent, respectively [42,43]. Equilibration involved an energy minimization, followed by 25 ps of NVT dynamics to reach a temperature of 300 K, and finally 475 ps NPT dynamics at 300 K under 1 atmosphere to finally obtained a stable density around 1.03 and a cubic box size of 58.6930534 51.1747184 47.6285708 for water and a stable density around 1.46 and a cubic box size of 58.6548076 51.2210897 47.4827615 Å for the CHCl_3_ solvent. Then, these data were used to perform molecular dynamics in the NVT ensemble at 300K during 160 ns, where the first 10 ns were taken as the additional equilibration and the remaining 150 ns as the production run**.**

#### 4.2.2. Analysis Trajectories

The trajectories were analyzed using cpptraj, the program processing amber trajectories files [44]. We then extracted data on hydrogen bonds, distances, and dihedral angles, and performed RMSD (root-mean-square deviation) type calculations. To define the existence of a hydrogen bond, the default values of cpptraj were always used, i.e., the distance cutoff from the acceptor to the donor heavy atom was 3.00 Å and the angle cutoff was set to 135°.

#### 4.2.3. Metadynamics in CHCl_3_/Water

Initialization: the system was first constructed with the aid of the PACKMOL software package [45] and consisted of two joined boxes of dimension 40 × 40 × 40 Å containing 1000 chloroform molecules and 4800 water molecules, respectively. The solute was placed at the center of the CHCl_3_ box. Forcefield parameters for the simulation were then built in the usual fashion using tleap: the TIP3P water model [42] was used as well as the chloroform model provided by the AMBER library *solvents.lib* [29]. Parameters for the solute were taken from GAFF [41] with partial charges obtained using the standard RESP fitting protocol. The system was minimized, and allowed to equilibrate at 300K for 25 ps in the *NVT* ensemble. Further equilibration in the *NPT* ensemble was carried out for 1.5 ns, with the overall density averaging around 1.2313 (see the Supplementary Materials Figures S12–S14 for equilibration data). Berendsen’s barostat was used to regulate the pressure with isotropic position scaling, a target pressure of 1.0 bar, and a pressure relaxation time of 2.0 ps. The Langevin thermostat was used to regulate the temperature, with a target temperature of 300 K and collision frequency of 5 ps-1. All other parameters were kept at their default values as implemented in Amber18.

Metadynamics: the simulation was run at 300 K in the *NVT* ensemble with a timestep of 2 fs, a friction coefficient of 1.0 ps^−1^, and a cutoff of 10.0 Å. The SHAKE algorithm was used to constrain hydrogen-containing bonds [46,47]. The open-source PLUMED library version 2.6.0 [48,49,50] was used to drive the metadynamics simulation. The distance between the center of masses of the solute and the chloroform box was computed and projected along the x axis (orthogonal to the CHCl_3_-H_2_O interface), constituting our collective variable. The well-tempered algorithm was used with a bias factor of 10.0, a hill height of 1.0 kJ.mol^−1^, and a hill width of 1.0 Å. Hills were deposited every 50,000 steps, corresponding to a 100 ps pace. The simulation was run for a total of 1135 ns, at which point the resulting PMF did not show any significant change and sufficient sampling of the collective variable was observed.

## Figures and Tables

**Table 1:** Inhibition of *T. gondii* intracellular growth of RH strain tachyzoite and human cells HFF with compound **1** compared to the natural product FR235222.

Cyclopeptides	*T. gondii* EC50 (nM)	HFF EC50 (nM)
FR235222	7.6 ± 0.6	107 ± 18
**1**	43 ± 2.2	280 ± 20

**Table 2:** Compound **1** efficiency on RH *Tg* HDAC3 T99A strain and on an RH wild-type strain compared to FR235222 (positive control) and DMSO (negative control). +: Tachyzoite proliferation leading to HFF cell lysis in cell culture observed by inverted microscopy. −: No tachyzoites proliferation with no cell lysis in cell culture observed by inverted microscopy.

	FR235222 (90 nM)	Compound 1 (90 nM)	DMSO
RH T99A	+	+	+
RH Wild Type	−	−	+

**Table 3:**
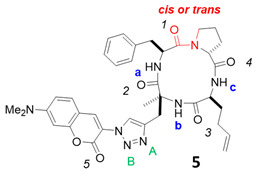
Percentage of the presence of hydrogen bond (Hbond) between water and compound **5** during the simulation.

Title 1	Water	0 Hbond	1 Hbond	2 Hbonds	3 Hbonds	Total
O**2**	H/donor	6%	51%	42%	1%	138%
O**3**	H/donor	6%	54%	39%	1%	135%
O**1**	H/donor	12%	65%	22%	1%	112%
O**5**	H/donor	15%	60%	24%	1%	111%
O**4**	H/donor	13%	69%	18%	0%	105%
N-H**a**	O/acceptor	55%	45%	0%	0%	45%
N**A**	H/donor	60%	37%	3%	0%	43%
N**B**	H/donor	72%	27%	1%	0%	29%
N-H**c**	O/acceptor	76%	24%	0%	0%	24%

## Data Availability

Data are available on request.

## Supplementary Materials

The following are available online, Figures S1–S7 are NMR data for compound **4**, Figure S8 represents superimposed MD data, Figures S9–S11 are representative structures of compound **5**. Figures S12–S14 show the evolution of system paramters during equilibration in the NPT ensemble. Figure S15 represents computed hydrophilic/hydrophobic surfaces for conformations of 5 in chloroform and in water.

## Sample Availability

Samples of the compounds **1**–**4** are available from the authors.
