# Supplementary material for: Crossing of the Cystic Barriers of Toxoplasma gondii by the Fluorescent Coumarin Tetra-Cyclopeptide
Source: Molecules. 2021 Dec 11;26(24):7506. doi: 10.3390/molecules26247506 (PMC8708940; doi:10.3390/molecules26247506)
Supplement: Supplementary file 1 [file molecules-26-07506-s001.zip › molecules-1480662-supplementary.pdf]

# Supplementary Material

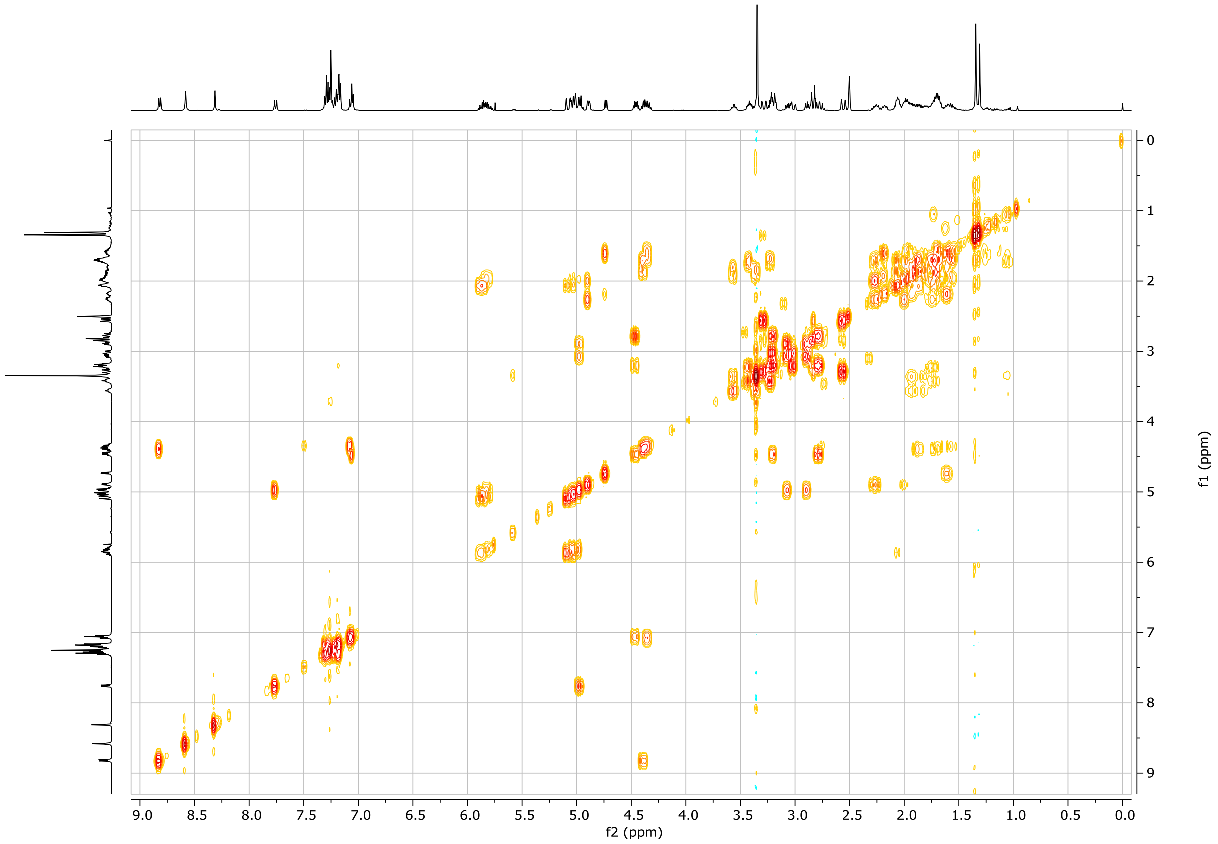


**Figure S1**. Compound **4** COSY in DMSO-*d6* (500 MHz)

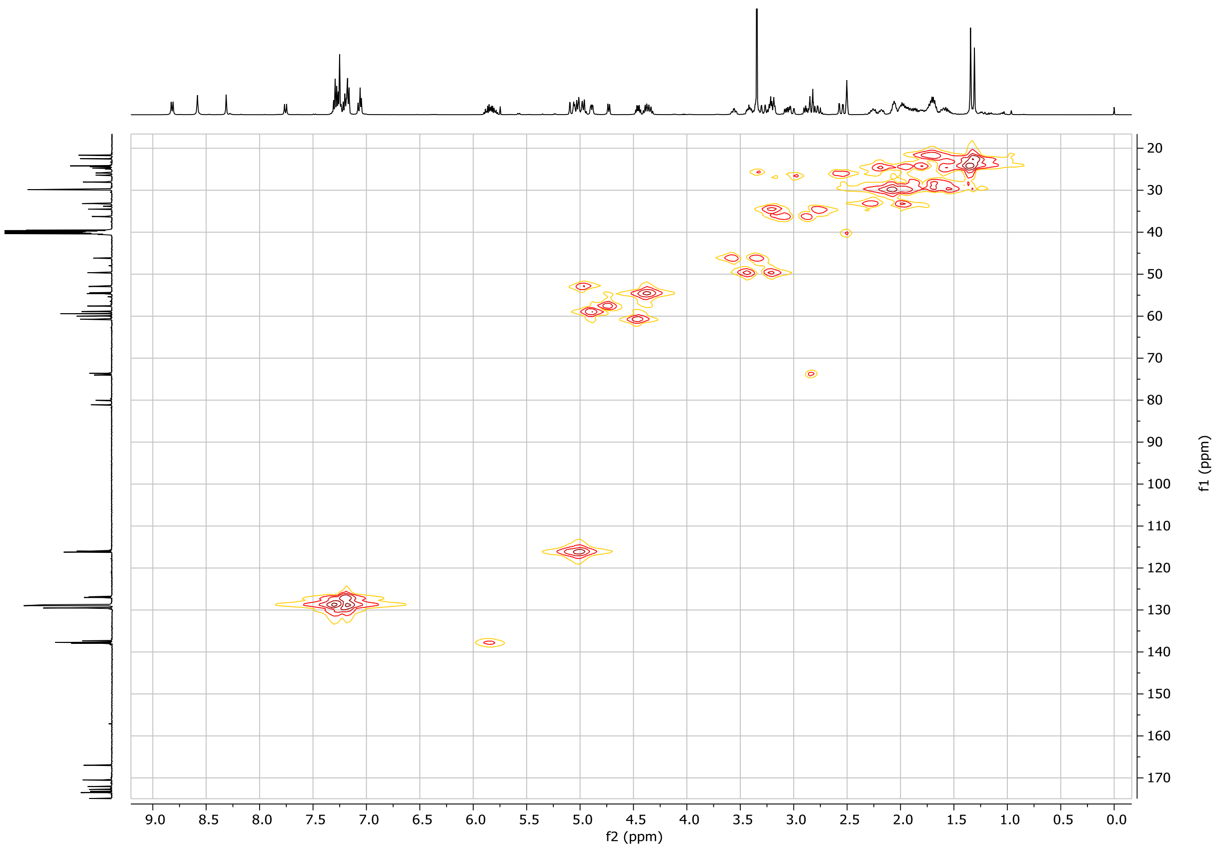


**Figure S2**. Compound **4** HMQC in DMSO-*d6* (500 MHz)

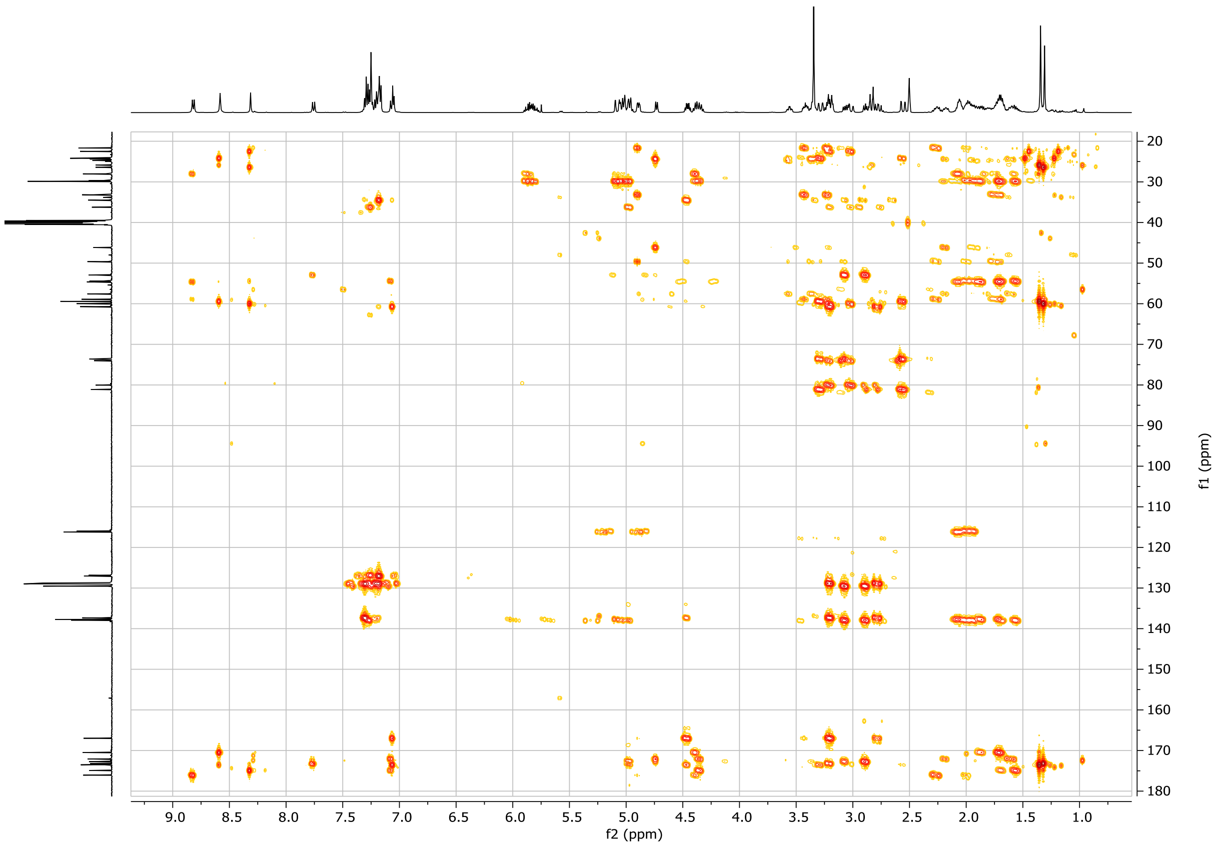


**Figure S3**. Compound **4** HMBC in DMSO-*d6* (500 MHz)

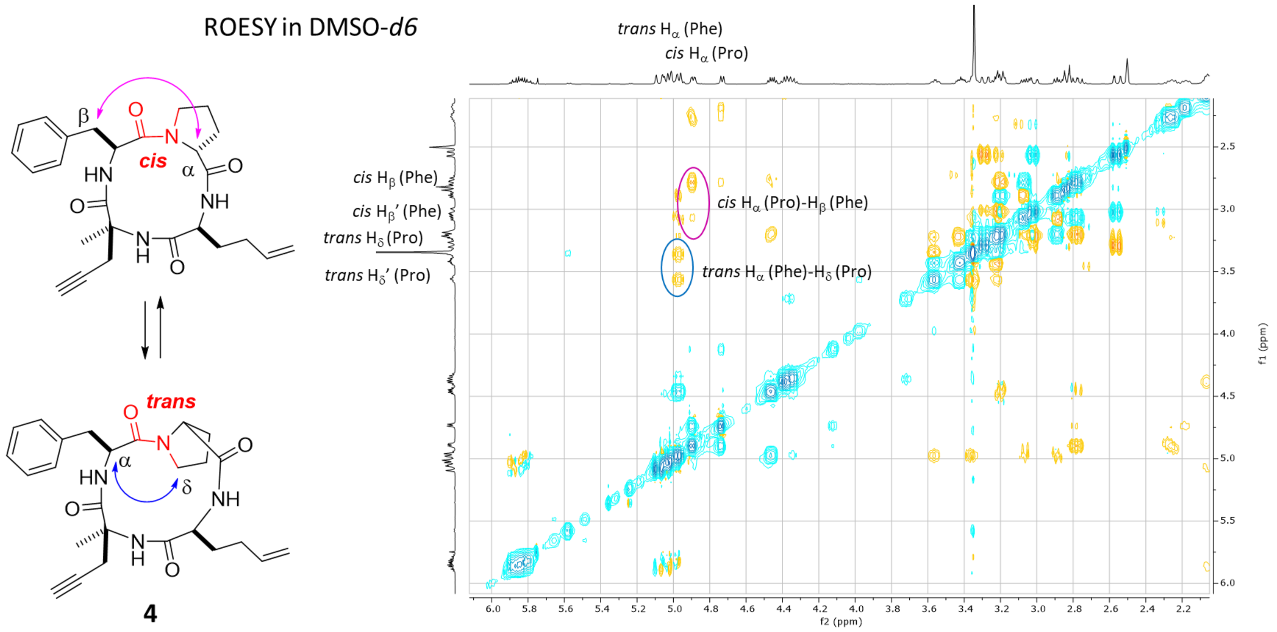


**Figure S4**. Compound **4** ROESY (mixing time 200 ms) in DMSO-*d6* (500 MHz)

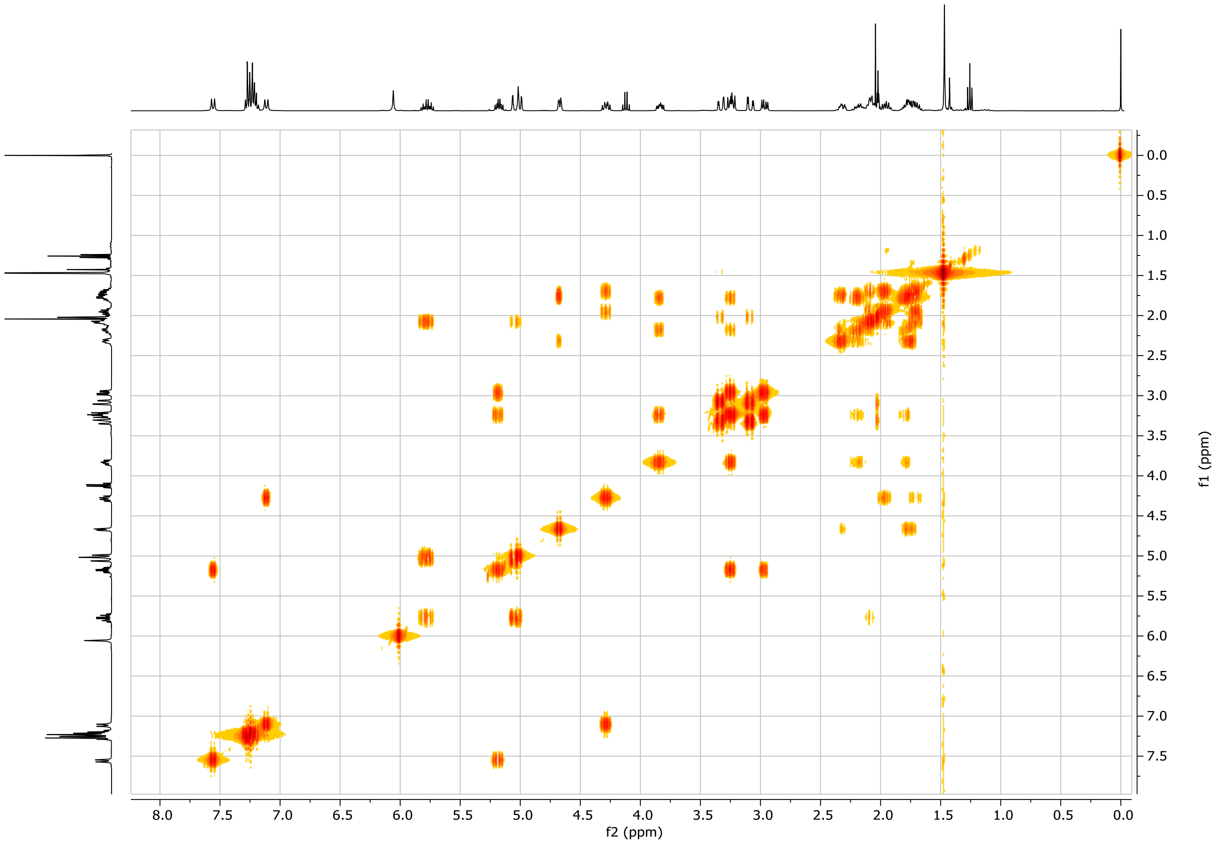


**Figure S5**. Compound **4** COSY in $CDCl_3$ (400 MHz)

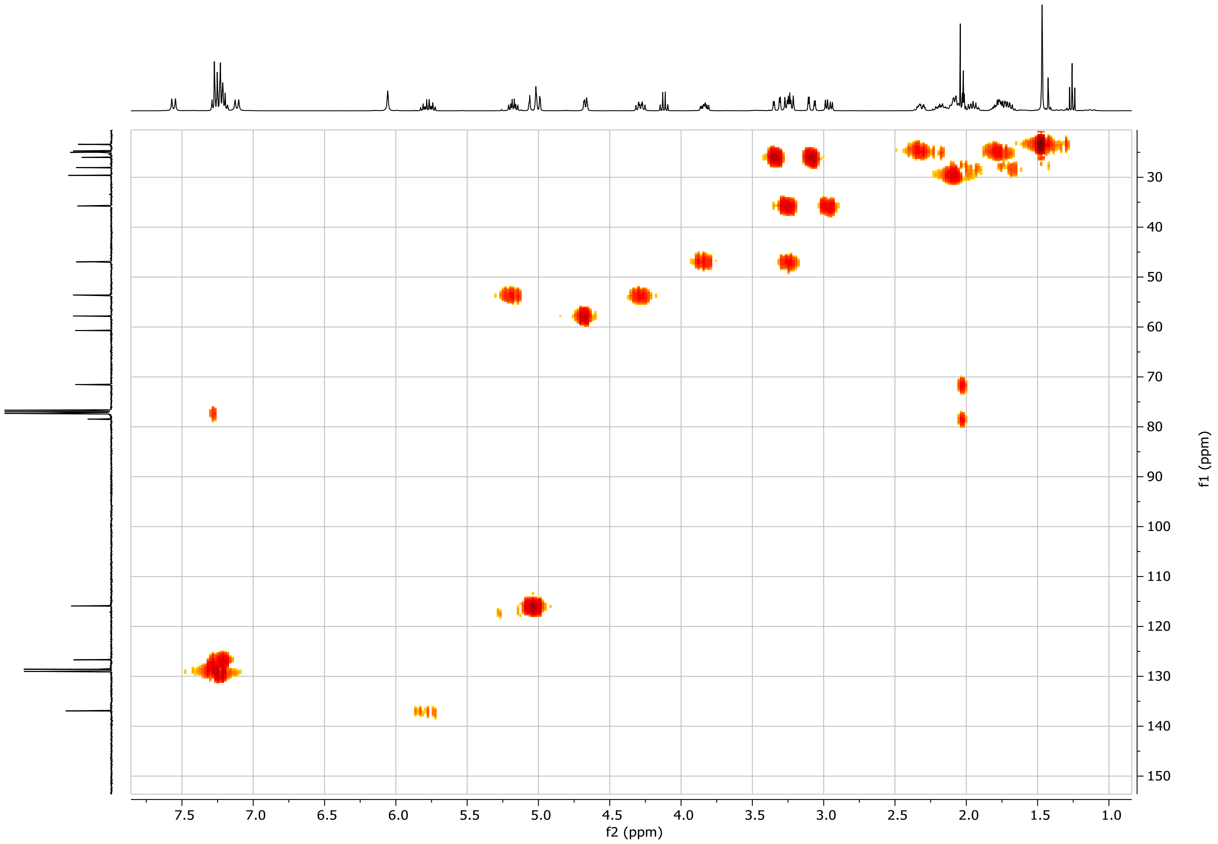


**Figure S6.** Compound **4** HMBC in $CDCl_3$ (400 MHz)

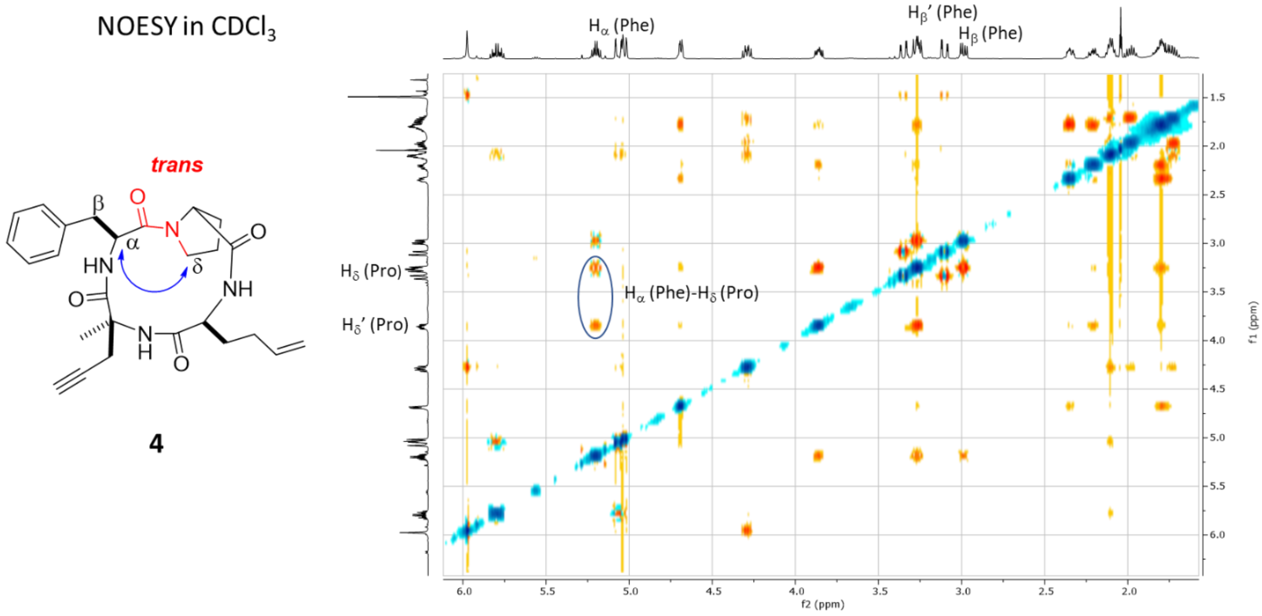


**Figure S7.** Compound **4** NOESY in $CDCl_3$ (400 MHz)

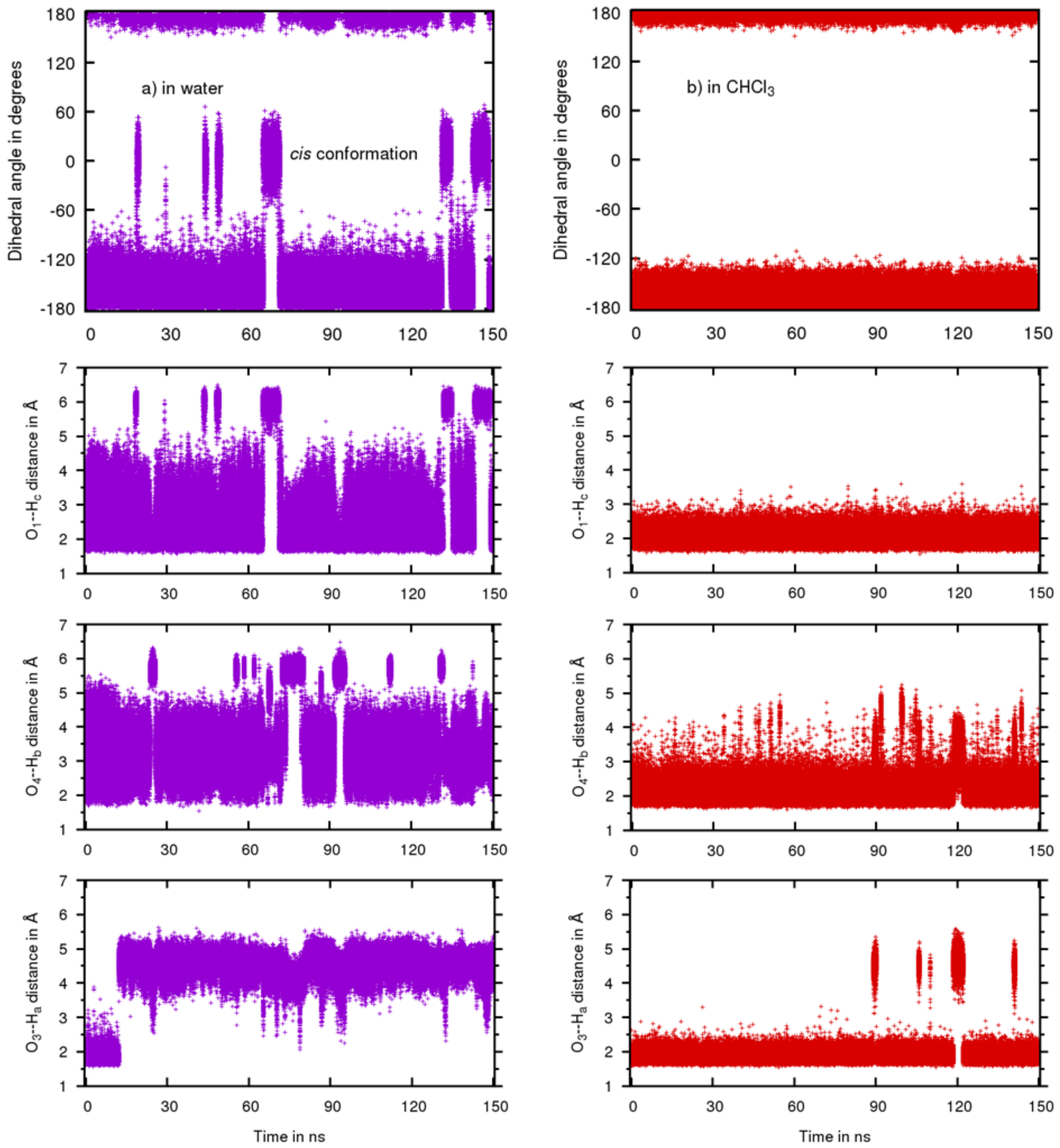


**Figure S8.** Molecular dynamics of compound **5** a) in water (cyan) and b) in $CHCl_3$ (red). The graphics of the dihedral angle between the proline and the phenylalanine (*cis*/*trans* isomerization) are aligned with the data of the hydrogen bond distance between C=O and NH.

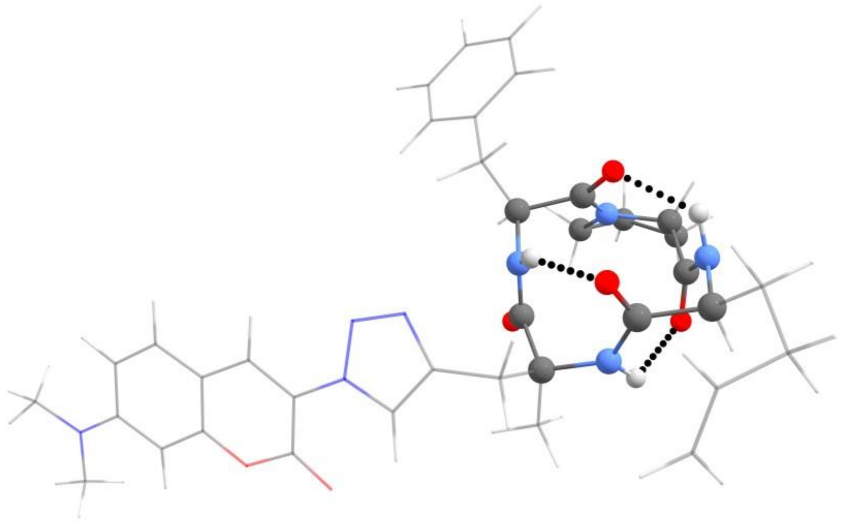

**Figure S9.** One structure of **5** in *trans* amide conformation between proline and phenylalanine in $CHCl_3$ during MD calculation

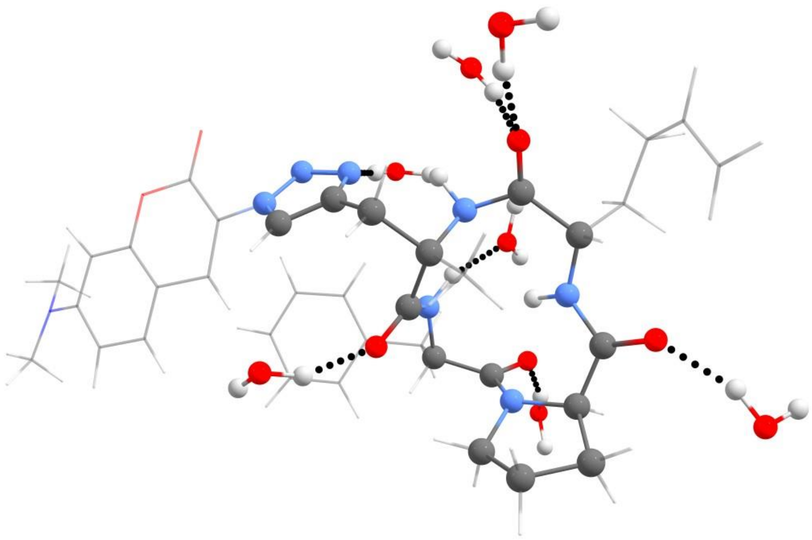

**Figure S10.** One structure of **5** in *trans* amide conformation between proline and phenylalanine in water during MD calculation

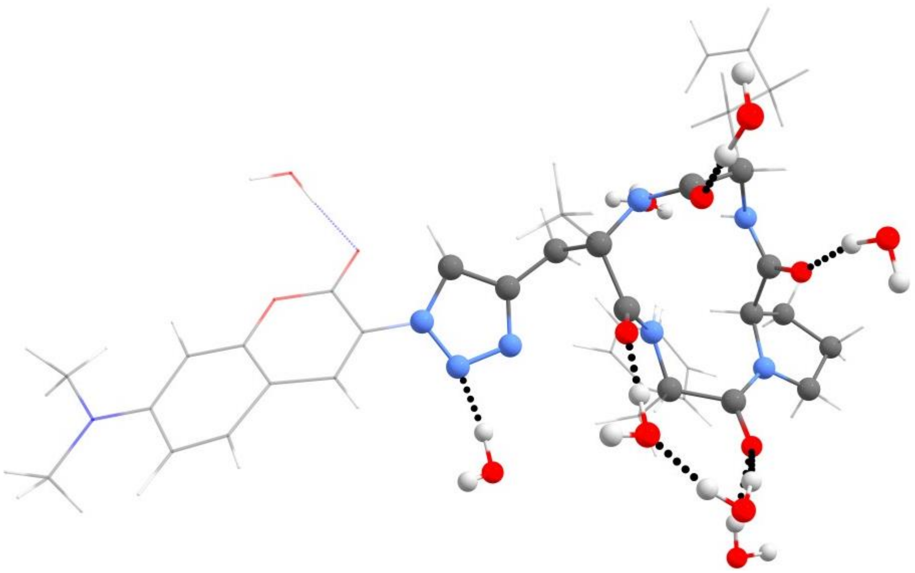

**Figure S11.** One structure of **5** in *cis* amide conformation between proline and phenylalanine in water during MD calculation

__Metadynamics system equilibration__

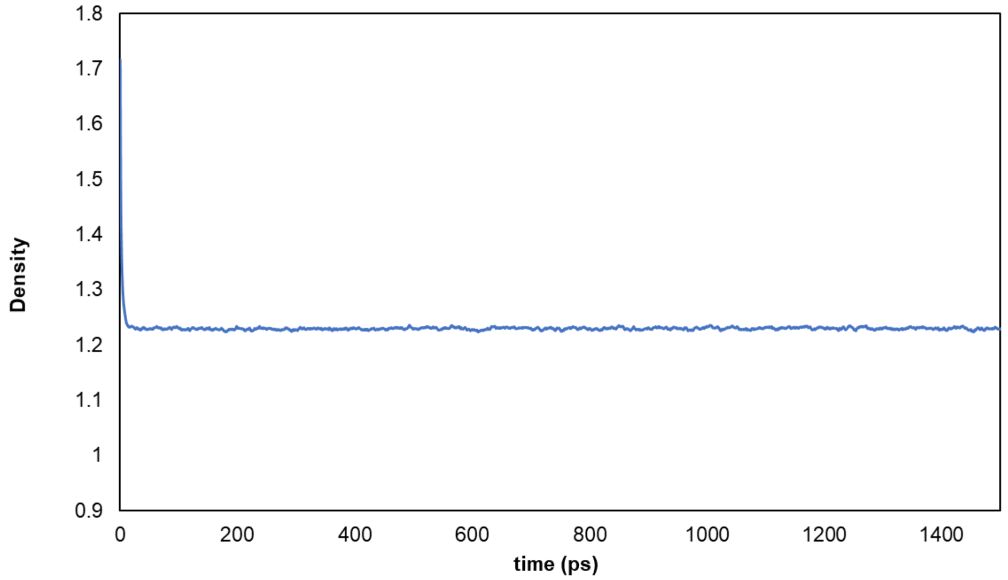


**Figure S12.** Evolution of the system density with simulation time (in ps) during the 1.5 ns equilibration run.

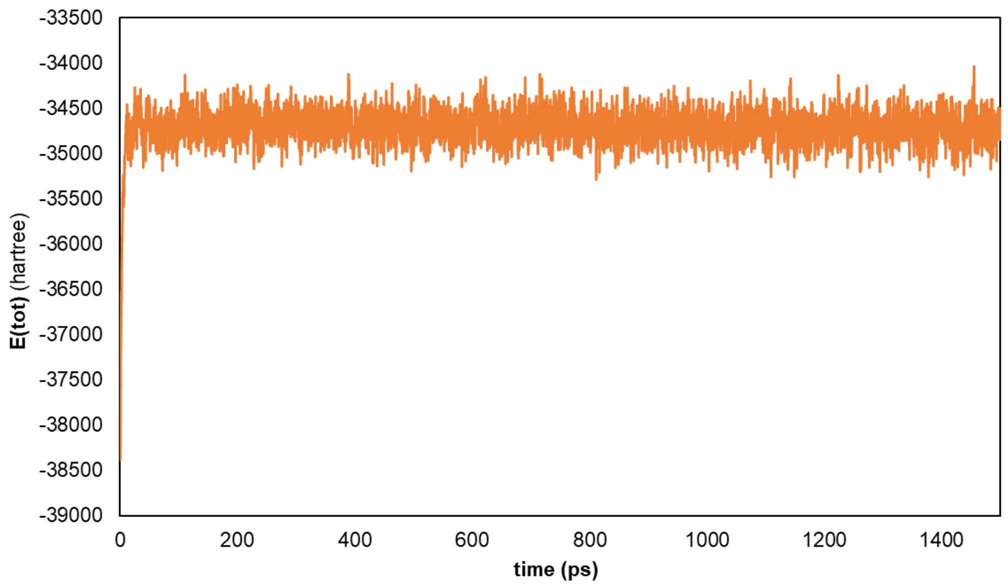


**Figure S13.** Evolution of the system's total energy (in hartree) with simulation time (in ps) during the 1.5 ns equilibration run.

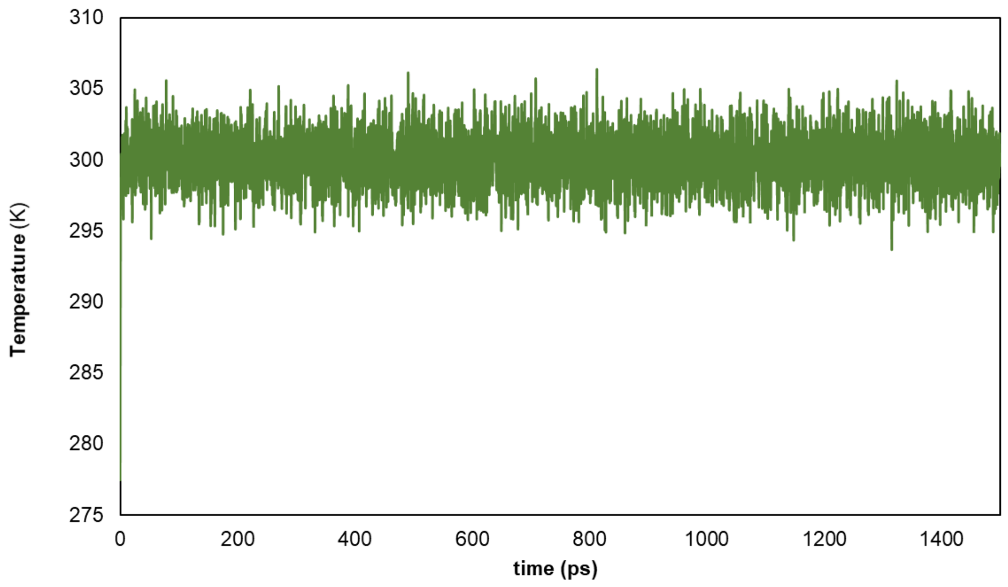


**Figure S14.** Evolution of the system's temperature (in K) with simulation time (in ps) during the 1.5 ns equilibration run.

**Hydrophilic / Hydrophobic surface areas**

Two conformations of compound **5** were taken from MD runs in $CHCl_3$ and water, whereby **5** prevalently features intramolecular H-bonding interactions and intermolecular H-bonding interactions, respectively. For these two conformations, a single point energy calculation was carried out in order to compute the electrostatic potential and project it onto the electron density surface.

The hydrophobic zones can then be visualized as the areas where the potential approaches zero whilst hydrophilic zones will be located in areas of high potential value (Scarsi et. al. Proteins: Struct. Funct. Genet. 2000, 37, 4, 565-575). The results are shown in Figure S15 below.

While the results do not show a significantly larger hydrophilic surface area in the "in water" conformation in comparison to the "in chloroform" conformer, the hydrophilic area patches around the cyclopeptide moiety are spread out over a wider area in the former as a result of its conformational flexibility induced by intermolecular hydrogen bonding interactions.

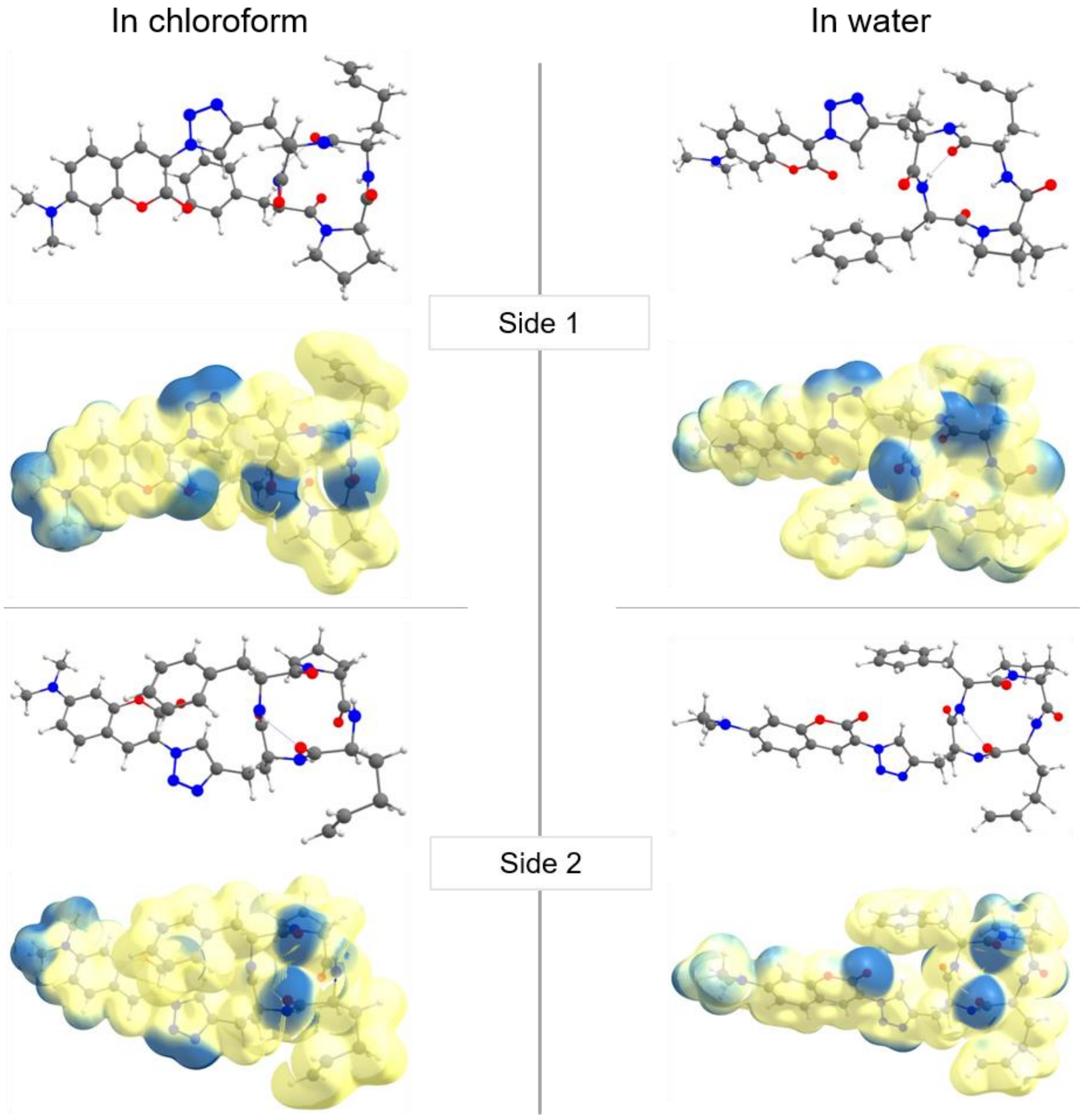


**Figure S15.** Comparison of hydrophobic (pale yellow) and hydrophilic (dark blue) surfaces, projected onto the electron density surface of compound **5** (isovalue = 0.003). Left, a conformation from the run in $CHCl_3$; Right, a conformation from the run in water.
